# Effect of Hydrogen Charging Current Density on Hydrogen Trapping Behavior in Cu6.01Ni2.7Mn Steel

**DOI:** 10.3390/ma19081521

**Published:** 2026-04-10

**Authors:** Wenxue Wang, Jing Guo, Jian Zhang, Lili Li

**Affiliations:** 1 School of Materials and Metallurgy, University of Science and Technology Liaoning, Anshan 114051, China; 15235926057@163.com (W.W.); guojing_neu@163.com (J.G.); 2 Fushun Special Steel Co., Ltd., Fushun 113000, China; zj2465996443@163.com

**Keywords:** copper-bearing steel, hydrogen embrittlement, hydrogen permeation, internal friction

## Abstract

Copper-containing steel is widely used in ship plates and other marine engineering fields due to its excellent mechanical properties and good weldability. However, in hydrogen-containing media environments, ship plate steel is prone to hydrogen embrittlement during service. Existing research primarily focuses on steel grades with copper content below 3 wt.%, while the diffusion and trapping behavior of hydrogen in ultra-high-copper steel with copper content exceeding 3 wt.% remains unclear. Therefore, this study designed an ultra-high-copper-content steel with a copper content of 6.01% and investigated the diffusion behavior of hydrogen in the test steel under different hydrogen charging current densities through microstructure characterization, slow strain rate tensile testing, electrochemical hydrogen permeation, and internal friction tests. The results indicate that with an increase in hydrogen charging current density, accompanied by a slight degradation in mechanical properties, the irreversible hydrogen trap density increases by 50.7%. A large number of microstructures, such as phase boundaries, grain boundaries, and dislocations, have formed inside the material, which have reversible trapping effects on hydrogen, effectively suppressing the migration of hydrogen in the crystal structure and reducing the embrittlement phenomenon caused by hydrogen. This study expands the application potential of copper-containing steel in the field of ocean engineering, providing an important reference for the future development of high-strength, hydrogen embrittlement-resistant copper steel with ultra-high copper content.

## 1. Introduction

Against the backdrop of persistently rising global energy demand and the gradual depletion of terrestrial oil and gas resources through exploration and development, the exploitation of offshore oil resources has become an imperative for ensuring national energy security [1,2]. However, marine environments are typically characterized by extreme conditions such as high corrosiveness, high pressure, and complex loads [3], which impose stringent requirements on the long-term in-service performance of marine engineering structural materials. Currently, high-strength low-carbon steels demonstrate significant application potential in marine engineering owing to their high strength, toughness, and corrosion resistance [4]. Compared to ordinary high-strength low-carbon steels, copper-bearing low-carbon steels, leveraging the ability of copper to optimize the structure of rust layers, can significantly enhance corrosion resistance while minimally affecting mechanical properties. Consequently, they are gradually emerging as key materials for deep-sea and offshore energy development equipment [5,6].

Copper-bearing low-carbon steels are in prolonged contact with seawater during service. Under hydrogen-rich conditions, hydrogen atoms enter the metal lattice via interstitial diffusion mechanisms and preferentially accumulate at defect sites such as grain boundaries and dislocations [7,8,9]. This hydrogen-induced damage can significantly degrade the mechanical properties of the steel and reduce its resistance to hydrogen embrittlement, thereby shortening the service life of engineering components [10,11,12,13]. This phenomenon is referred to as hydrogen embrittlement (HE). Strategies to enhance a material’s resistance to HE include the effective introduction of hydrogen traps [14,15,16], grain boundary engineering [17], and microalloying [18]. The mechanism of hydrogen-induced brittle failure primarily involves three stages: hydrogen permeation, hydrogen atom migration, and the nucleation and propagation of hydrogen-induced cracks [19,20,21,22]. The nucleation and propagation of hydrogen-induced cracks can lead to a localized transition from ductile to brittle behavior in the material, resulting in the loss of its original functionality. Therefore, an in-depth investigation into the hydrogen embrittlement mechanisms of copper-bearing low-carbon steels is of critical importance.

A systematic experimental research framework has been established within the academic community for investigating the mechanisms of hydrogen embrittlement. Among these, hydrogen charging current density is widely studied as a key external variable to evaluate the hydrogen embrittlement resistance of different steels. Dan, W.J. [23] systematically investigated the mechanical response of Q690 steel under varying hydrogen charging parameters. The experimental results indicated that electrochemical hydrogen charging parameters significantly affect the material’s mechanical behavior: mechanical properties showed a linear decreasing relationship with hydrogen charging current density, while exhibiting nonlinear attenuation trends with charging time and solution concentration. As the hydrogen charging parameters increased, the tensile strength and yield stress decreased by 2–4%, the elongation after fracture decreased by up to 20%, and macroscopic necking gradually diminished until disappearing. Fractographic analysis revealed that as hydrogen ingress increased, the number of dimples decreased significantly, and the fracture mechanism transitioned from ductile to completely brittle. This study elucidated the quantitative relationship between hydrogen ingress parameters and the degradation of material mechanical properties. Xu, C.J. [24], through electrochemical cathodic hydrogen charging experiments, systematically investigated the effects of hydrogen charging current density and temperature on the material’s hydrogen permeation behavior and embrittlement sensitivity. The results showed that both hydrogen permeation rate and hydrogen diffusion coefficient increased significantly with higher charging current density and temperature. The tensile plasticity of the material exhibited an accelerated degradation trend as the hydrogen charging current density increased, with the hydrogen embrittlement index reaching its peak at 308 K. Fracture surfaces displayed brittle characteristics, including quasi-cleavage facets and microcracks, revealing the coupled effect mechanism of hydrogen environmental parameters on the hydrogen embrittlement behavior of Cr-Mo steels. Peral, L.B. [25], through electrochemical hydrogen permeation experiments, systematically examined the effects of different solution compositions, hydrogen charging current densities, and surface roughness on the hydrogen permeation behavior of quenched and tempered low-alloy steels. The experiments found that hydrogen transport was dominated by lattice diffusion in acidic media containing poisoning agents under high-current-density hydrogen charging, whereas in other media, hydrogen migration was primarily controlled by trapping–detrapping processes. Du, Y. [26] systematically explored the influence of heat treatment processes on the hydrogen embrittlement behavior of low-carbon medium-manganese steel. Experiments revealed that direct water-quenched samples exhibited higher hydrogen permeation rates due to their high dislocation density, while samples subjected to intercritical annealing effectively suppressed hydrogen embrittlement sensitivity by forming stable retained austenite. The study found that as the volume fraction of retained austenite increased, the material’s hydrogen embrittlement index significantly decreased, primarily due to Mn enrichment enhancing the stability of retained austenite and its hydrogen trapping capacity. Fractographic analysis showed that optimally treated samples exhibited a combination of quasi-cleavage and dimple features, confirming the hindering effect of retained austenite on hydrogen-induced crack propagation. Wang, Q.Y. [27] found that under hydrogen-rich conditions, laser-peened 316L stainless steel exhibited lower hydrogen absorption capacity compared to non-peened 316L stainless steel, while also enhancing its corrosion resistance. Comprehensive studies indicate that hydrogen charging current density is a key external factor driving hydrogen-induced embrittlement, but its effects are synergistically regulated by material microstructure, environmental media, and temperature. By optimizing microstructure or implementing surface modifications, hydrogen permeation and accumulation can be effectively mitigated.

Existing studies have confirmed the hydrogen embrittlement resistance of copper-containing steel in hydrogen-rich environments. However, the hydrogen diffusion and embrittlement behavior of ultra-high-copper steel under different hydrogen charging conditions remains insufficiently explored. Current research has a limitation in that copper content is generally low [28,29,30]. Nevertheless, it has been reported in the literature that Cu-rich nanoprecipitates can mitigate hydrogen embrittlement by trapping hydrogen at precipitate/matrix interfaces [31]. To address this, the present study designed and developed an ultra-high copper test steel with a copper content of 6.01%. This paper systematically investigates the hydrogen diffusion behavior, hydrogen-induced internal friction behavior, and hydrogen embrittlement susceptibility of ultra-high-copper steel under various hydrogen charging environments. It elucidates the influence of different hydrogen charging current densities on hydrogen trapping characteristics, hydrogen diffusion, and hydrogen embrittlement failure, and reveals the hydrogen diffusion mechanism in the steel. These findings provide theoretical support for the hydrogen trap design and engineering application of high-copper-content steel materials.

## 2. Materials and Methods

### 2.1. Experimental Materials

**Figure 1:**
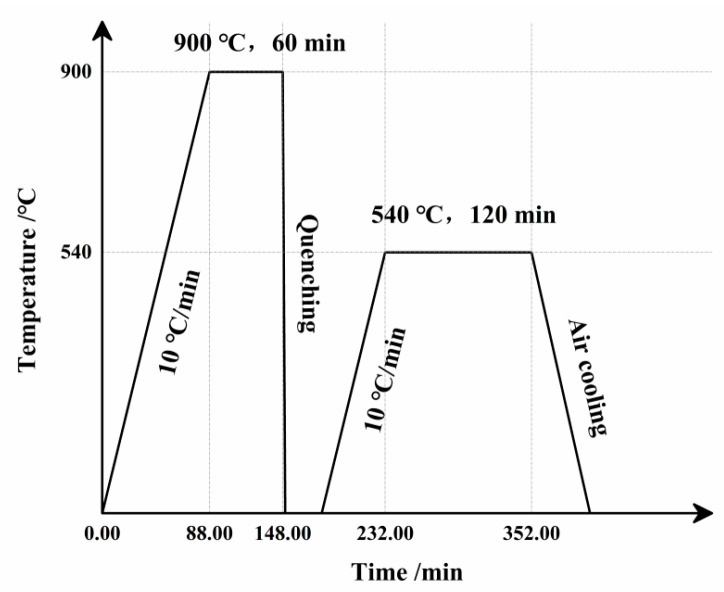
Heat treatment process curve of test steel.

The experimental steel was smelted using a vacuum induction furnace. Considering the relatively high melting point of copper, the steel was heated in a soaking furnace to 1100 °C and held for 4 h before rolling. Hot rolling was performed to obtain steel plates with final dimensions of 400 mm × 100 mm × 20 mm. The chemical composition of the steel is listed in Table 1. Figure 1 illustrates the heat treatment process for the experimental steel, which consisted of the following steps: heating from room temperature to 900 °C at a rate of 10 °C/min, holding at 900 °C for 60 min, followed by water cooling; then reheating to 540 °C at 10 °C/min, holding for 120 min, and finally air cooling to room temperature.

### 2.2. Experimental Methods

For material characterization, the metallographic specimens were sequentially ground, mechanically polished, and etched using a 4% nital solution. The microstructure was subsequently observed under a Zeiss Axio VERT.A1 optical microscope (Carl Zeiss Microscopy GmbH, Oberkochen, Germany).

The hydrogen permeation behavior of the experimental steels was investigated using an Fe-HP-12 metal hydrogen permeation testing apparatus (ACM Instruments, Grange-over-Sands, UK) to study hydrogen diffusion in the different steels. Samples for hydrogen permeation testing were prepared with dimensions of 80 mm × 50 mm × 1 mm. After cutting, the specimens were mechanically ground to a standard thickness of 1.0 ± 0.02 mm. The ground samples were then subjected to constant-current electrochemical polishing in a phosphoric acid-based electrolyte (70% H_3_PO_4_, 10% H_2_SO_4_, 9% CrO_3_, 11% H_2_O) at a constant current of 3.5 A for 4 min, followed by thorough rinsing with deionized water. Subsequently, the specimens were cathodically activated in a 10 wt.% HCl solution at a constant current of 0.7 A for 15 s, immediately washed with deionized water, and then electroplated with nickel on one side under a constant current of 70 mA for 60 s. This prepared the specimens for the hydrogen permeation experiments.

**Figure 2:**
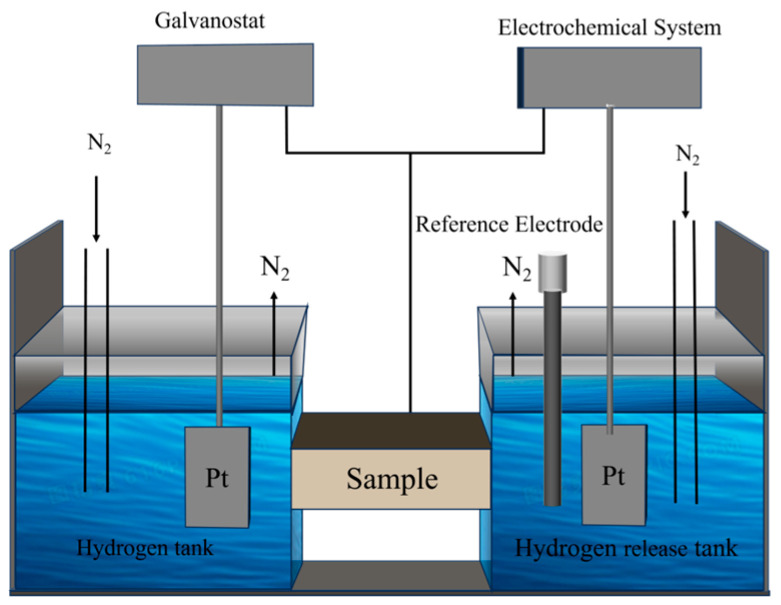
Schematic diagram of hydrogen permeability tester.

The pretreated hydrogen permeation specimen was placed in the center of the electrolytic cell setup. Throughout the two hydrogen permeation tests, nitrogen gas was continuously introduced into both electrolytic cells at a flow rate of 10 mL/min for deoxygenation. The electrolyte solution consisted of 0.5 mol/L NaOH with 3 mL of thiourea. The hydrogen charging duration was 8 h, with charging current densities set at 10 mA/cm^2^, 15 mA/cm^2^, and 20 mA/cm^2^, respectively, to investigate the permeation behavior of hydrogen in the material under different charging intensities. A schematic diagram of the experimental setup is shown in Figure 2.

**Figure 3:**
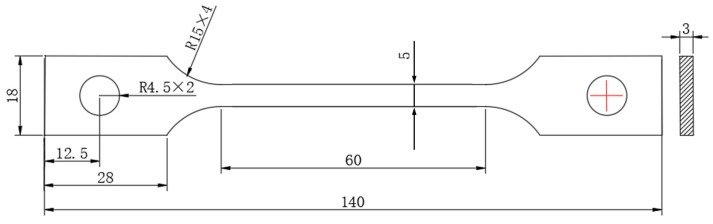
Tensile standard sample at room temperature.

Tensile tests were conducted using a Shimadzu AG-X universal tensile testing machine (Shimadzu, Kyoto, Japan). Tensile specimens were prepared according to the national standard GB/T 228.1-2021 “Metallic Materials—Tensile Testing—Part 1: Method of Test at Room Temperature” [32], utilizing sheet tensile specimens with a thickness of 3 mm, and the specific dimensions of the tensile specimen are shown in Figure 3. Slow strain rate tensile tests at a speed of 0.018 mm/min were then performed on the experimental steel both before and after hydrogen charging to evaluate its hydrogen embrittlement sensitivity. All reported data represent the average of three repeated tests.

For the internal friction experiments, an MFP-1000 internal friction tester (Institute of Solid State Physics, Hefei, China) was used. Specimens were machined via wire cutting into standard dimensions of 50 mm × 2.08 mm × 0.98 mm. Hydrogen charging was performed in an electrochemical hydrogen permeation setup using gradient current densities of 10 mA/cm^2^, 15 mA/cm^2^, and 20 mA/cm^2^ for 8 h each. The electrolyte solution consisted of 0.5 mol/L NaOH with 3 mL of thiourea. Subsequently, the energy dissipation characteristics of the specimens before and after hydrogen permeation were measured using the free-decay vibration method over a temperature range of −120 °C to 400 °C.

## 3. Results

### 3.1. Microstructure

**Figure 4:**
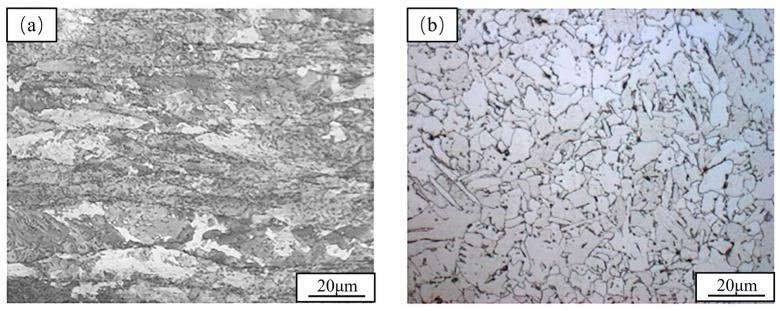
Microstructure of test steel: (**a**) As-rolled, (**b**) Heat-treated.

Figure 4 shows the transverse original state and metallographic structure of the test steel after heat treatment. From Figure 4a, it can be seen that the microstructure of the test steel was mainly composed of ferrite and granular bainite. However, the black stripes along the rolling direction indicated the presence of local strain bands during the rolling process, which had been caused by uneven temperature distribution and rolling force. For this reason, the heat treatment process had been carried out on the material. It could be seen from Figure 4b that the resulting structure had been mainly lath bainite, and a small amount of pearlite had also appeared. Compared with Figure 4a, not only had the black stripes disappeared, but the grains had also become more uniform. The grain size was quantitatively measured using the intercept method. The average grain size in Figure 4a is 5.3 *±* 0.8 μm, and in Figure 4b it is 8.6 *±* 1.2 μm. The statistical results show that the average grain size increases after heat treatment. However, this is not simple grain coarsening. It is closely related to the elimination of deformation bands and structural reconstruction. The deformation band regions in the original sample contain numerous sub-grain boundaries and high-density dislocations. These features refine the local grains and lower the overall average size. Recovery and recrystallization occur during heat treatment. The deformation bands disappear, and the grains grow uniformly. Although the average size increases, the structural uniformity improves significantly. The black deformation bands observed in this study are attributed to local stress concentration during the rolling process. These bands typically contain high-density dislocations and lattice distortion. Such regions act as favorable trapping sites for hydrogen. They also serve as preferential nucleation sites for hydrogen-induced cracking. In the original sample, deformation bands function as preferential aggregation zones for hydrogen atoms. After heat treatment, the deformation bands disappear and the grains become uniform. This eliminates the localized hydrogen traps. It thereby improves the hydrogen embrittlement resistance of the material.

### 3.2. Mechanical Properties and Hydrogen Embrittlement Sensitivity

**Figure 5:**
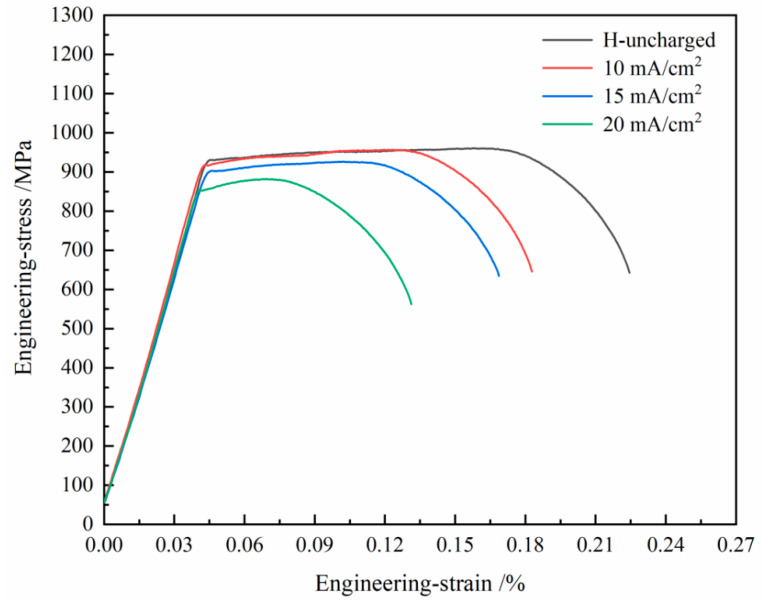
Engineering stress-engineering strain curves of test steel under different hydrogen charging current densities.

Figure 5 shows the engineering stress–strain curves of the experimental steel under different hydrogen charging current densities. The mechanical properties before and after hydrogen charging are shown in Table 2. From Figure 5, it can be seen that the material did not experience a sudden decrease in stress during the yielding stage, but showed a continuous increase accompanied by a long plateau, indicating a continuous yielding type and demonstrating stable, uniform plastic deformation ability. Its tensile strength and yield strength reached their maximum values simultaneously during this stage, and the material maintained its strength without significant decrease during the continuous deformation process. As the hydrogen charging current density increases, the yield plateau gradually shortens or even disappears, and the mechanical properties of the material show a significant degradation trend. When the current density increases to 10 mA/cm^2^, the yield plateau still exists. However, its corresponding yield strength decreases compared to the uncharged state. The tensile strength also decreases simultaneously. As the current density further increases to 15 mA/cm^2^, the yield plateau begins to shorten. Stress fluctuations in the plateau region become gentler. At the same time, the decrease in tensile strength and yield strength further expands. It is worth noting that at a high hydrogen charging current density of 20 mA/cm^2^, continuous yielding almost disappears, and the stress–strain curve rapidly decreases after reaching its peak. The rapid proliferation of dislocations and the formation of stress concentrations promote microcrack nucleation. This, combined with a high hydrogen charging current density, significantly weakens the material’s deformability in the plastic stage and triggers brittle fracture, consequently leading to a marked degradation in macroscopic ductility. The hydrogen embrittlement sensitivity (*I_HE_*) can be calculated based on the change in elongation of the sample before and after hydrogen charging, as shown in Equation (1).

(1) $$I_{\mathrm{HE}}=\left[\left(\varepsilon_{1}-\varepsilon_{2}\right)/\varepsilon_{1}\right]\;\times \;100\%$$

where *ɛ*_1_ is the elongation of the uncharged specimen; *ɛ*_2_ is the elongation of the hydrogen-charged specimen.

Under non-hydrogen-charged conditions, the experimental steel exhibited excellent comprehensive mechanical properties, with yield strength, tensile strength, and elongation of 916 MPa, 960 MPa, and 22.8%, respectively. When the hydrogen charging current density increased to 10 mA/cm^2^, the material showed significant hydrogen embrittlement effects, with varying degrees of degradation in mechanical properties. Specifically, yield strength and tensile strength slightly decreased by 0.76% and 0.4%, respectively, while elongation significantly dropped to 17.3%, a reduction of 24.1%. The hydrogen embrittlement sensitivity reached 24.1%, indicating that hydrogen atoms initially intruded rapidly and hindered dislocation movement, leading to a substantial loss of plasticity. When the hydrogen charging current increased to 15 mA/cm^2^, the degradation of material properties accelerated. Yield strength decreased to 888 MPa (a 3.0% reduction), tensile strength dropped to 926 MPa (a 3.5% reduction), and elongation decreased to 16.8%. Compared to the previous stage, the reduction in elongation was relatively smaller. At this stage, hydrogen damage entered an accumulation phase, with strength degradation becoming the dominant failure mode, while the loss of plasticity temporarily slowed. Under the highest hydrogen charging current density (20 mA/cm^2^), the material properties deteriorated comprehensively. Yield strength sharply decreased to 840 MPa (an 8.3% reduction), tensile strength dropped to 861 MPa (a 10.3% reduction), and elongation drastically fell to 13.1%. The hydrogen embrittlement sensitivity rose to 42.5%. At this point, with hydrogen embrittlement sensitivity exceeding 40%, the material entered a high-risk failure range. The significant decline in both strength and plasticity indicated that the high-current hydrogen environment had induced grain boundary embrittlement and crack propagation. Furthermore, the results show that both strength and plasticity decreased in a stepwise manner with increasing current. Yield strength, tensile strength, and elongation all exhibited a nonlinear decreasing trend with higher hydrogen charging currents, with the degradation rate significantly accelerating when the current density exceeded 15 mA/cm^2^.

**Figure 6:**
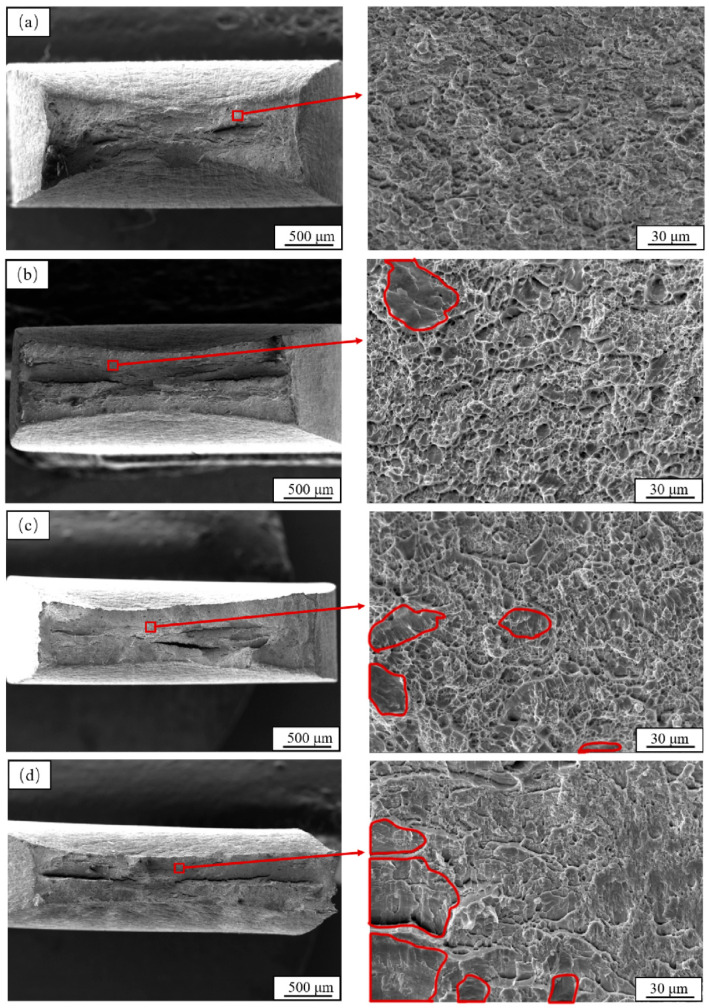
Fracture morphology of test steel under different hydrogen charging current densities: (**a**) uncharged with hydrogen, (**b**) 10 mA/cm^2^, (**c**) 15 mA/cm^2^, (**d**) 20 mA/cm^2^. The red circles indicate the brittle fracture regions.

Figure 6 shows the tensile fracture morphologies of the tested steel under different hydrogen charging current densities. Comparative analysis reveals that the hydrogen charging current density significantly influences the fracture behavior of the material. As the hydrogen charging current density increases, the brittle fracture area on the tensile fracture surface enlarges. In the uncharged condition, the fracture surface exhibits typical ductile fracture characteristics: macroscopically, the fracture appears cup-cone shaped with noticeable necking, while microscopically, the fracture surface is uniformly covered with numerous equiaxed dimples, indicating the material’s excellent plastic deformation capability in its original state. When the hydrogen charging current density increases to 10 mA/cm^2^, macroscopic necking remains relatively evident, and the microscopic fracture surface is still dominated by dimples. However, shallow cleavage facets appear in some regions, suggesting that hydrogen atoms have begun to diffuse and induce mild hydrogen embrittlement. At a higher charging current density of 15 mA/cm^2^, macroscopic observation reveals slight necking, while microscopic features show a significant decrease in dimple density and a marked increase in cleavage fracture proportion. This indicates that hydrogen accumulation at grain boundaries intensifies local embrittlement. At a current density of 20 mA/cm^2^, the fracture surface exhibits a mixed mode, characterized by increased proportions of cleavage facets and intergranular fracture, indicating that hydrogen-induced embrittlement dominates under these conditions.

Quantitative analysis of the brittle fracture area fraction under different hydrogen charging conditions was conducted, and the results are summarized in Table 3. For the uncharged specimen, no brittle fracture features were observed, corresponding to a brittle fracture area fraction of 0. At a hydrogen charging current density of 10 mA/cm^2^, the brittle fracture area fraction was approximately 5%, indicating that hydrogen-induced embrittlement remained relatively mild. When the current density increased to 15 mA/cm^2^, the brittle fracture area fraction rose to 12%, consistent with the marked increase in cleavage fracture observed in the microscopic morphology. As the current density further increased to 20 mA/cm^2^, the brittle fracture area fraction reached 30%, indicating that brittle fracture characteristics became dominant. These quantitative results are in good agreement with the fractographic observations, further confirming the significant influence of hydrogen charging current density on the hydrogen embrittlement susceptibility of the ultra-high-copper steel.

### 3.3. Hydrogen Diffusion Behavior

**Figure 7:**
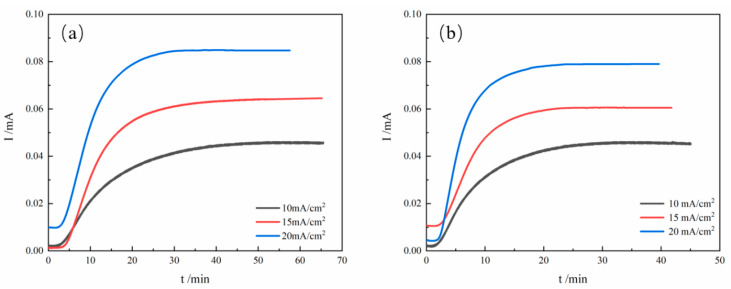
A comparison between the two permeation tests: (**a**) first hydrogen permeation, (**b**) second hydrogen permeation.

Figure 7 presents the anode-side transient current-time curves during hydrogen permeation as a function of charging current density. Table 4 summarizes the corresponding hydrogen permeation parameters. As shown in Figure 7, the transient current on the anode side exhibits an exponential growth characteristic in the initial stage, followed by a steady-state plateau region. As the current density increases, the permeation time shortens significantly, with the steady-state lag time decreasing from 862.2 s to 603 s. However, the steady-state current value shows a declining trend, dropping from 0.0845 mA to 0.046 mA.

The calculations for the effective hydrogen diffusion coefficient (*D_eff_*), hydrogen permeation flux (*J_ss_*), hydrogen concentration on the charging side of the specimen (*C*_0_), number of hydrogen traps per unit volume (*N_T_*), and number of irreversible traps per unit volume (*N_ir_*) are provided in Equations (2)–(6) [33].

(2) $$J_{SS}=\frac{i_{SS}}{AF}$$

(3) $$C_{0}=\frac{J_{SS}L}{D_{eff}}$$

(4) $$D_{eff}=\frac{L^{2}}{{6t}_{lag}}=D_{L}\left(1+\frac{{3N}_{T}}{N_{A}\cdot C_{0}}\right)$$

(5) $$N_{T}=\frac{N_{A}\cdot C_{0}}{3}\left(\frac{D_{L}}{D_{eff}}-1\right)$$

(6) $$\begin{array}{c} N_{ir}=N_{T1}-N_{T2} \end{array}$$

where *i_ss_* is the steady-state hydrogen permeation current density, representing the hydrogen permeation rate, in mA/cm^2^; *A* is the effective interfacial area between the specimen and the electrolyte, in cm^2^; *L* is the thickness of the hydrogen charging zone, 1 mm; *F* is Faraday’s constant, 96,485.3 C/mol; *t_lag_* is the time corresponding to *i*/*i_s_* = 0.63, in s, where *i* is the instantaneous current during hydrogen permeation; *D_L_* is the lattice diffusion coefficient, 9.5 × 10^−5^ cm^2^/s; *N_A_* is Avogadro’s number, 6.02 × 10^23^; *C_OR_* is the concentration of reversible hydrogen traps, in mol/cm^3^; *D*_1_ is the lattice diffusion coefficient of the α-Fe matrix, 1.28 × 10^−4^ cm^2^/s; *N_T_*_1_ and *N_T_*_2_ are the trap densities from the two permeation experiments, in cm^−3^.

Table 4 compares the hydrogen permeation parameters under the three current densities. It can be observed that the lag time (*t_lag_*) for hydrogen atoms to penetrate the specimen exhibits a decreasing trend, declining from 862.2 s to 603.6 s. The increase in hydrogen concentration accelerates hydrogen injection and diffusion, leading to faster penetration through the material. Additionally, the penetration time in the second hydrogen permeation test is generally lower than that in the first, attributed to partial saturation of traps in the pre-charged material, which reduces hydrogen retention time. Both the hydrogen permeation flux (*J_ss_*) and the effective hydrogen diffusion coefficient (*D_eff_*) show significant increasing trends. A comparison between the two permeation tests reveals that the *D_eff_* values in the second permeation stage are markedly higher than those in the first. This is primarily due to irreversible hydrogen traps within the material, including stable trapping sites formed by crystal defects such as grain boundaries and dislocations. These microstructural features immobilize hydrogen atoms through strong binding energies, altering their diffusion kinetic pathways. The hydrogen trap density (*N_T_*) increases from 3.36 × 10^22^ cm^−3^ to 8.97 × 10^22^ cm^−3^, and the irreversible hydrogen trap density (*N_ir_*) also shows a notable increase after hydrogen charging, rising from 2.7 × 10^21^ cm^−3^ to 4.76 × 10^22^ cm^−3^. This is mainly attributed to the synergistic effect of elevated hydrogen concentration and dynamic microstructural evolution. Under high current densities, the increased hydrogen flux leads to hydrogen supersaturation, activating reversible traps such as dislocations and grain boundaries while promoting the binding of hydrogen atoms to irreversible traps, such as precipitate interfaces. As the hydrogen charging current density increases, the driving force for hydrogen atom diffusion increases, and the diffusion coefficient rises significantly. This enhanced driving force allows more hydrogen traps in the steel to be occupied, leading to an increase in the number of hydrogen traps. At low hydrogen charging current densities, the number of hydrogen atoms entering the steel is limited. When the hydrogen charging current density increases, the hydrogen concentration in the steel rises substantially. At this stage, not only are strong hydrogen traps filled, but a large amount of hydrogen also begins to occupy traps with lower binding energies. A high hydrogen charging current density signifies an extremely high cathodic overpotential. This leads to a very high concentration of hydrogen adsorbed on the material surface. Consequently, enormous hydrogen pressure is generated in the subsurface region. This pressure induces new plastic deformation in localized areas, especially at pre-existing microscopic defects. It thereby creates new hydrogen traps, such as dislocations, microcracks, or microvoids.

### 3.4. Internal Friction Behavior

**Figure 8:**
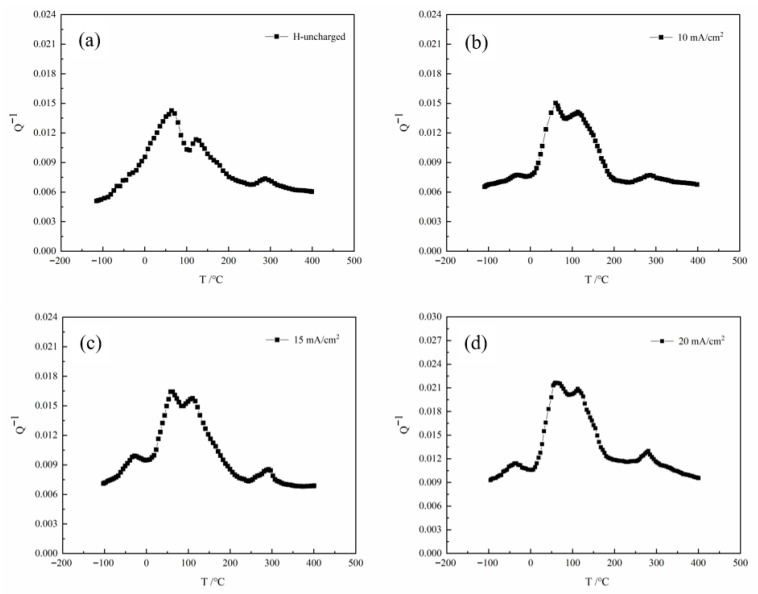
Internal consumption spectrum of different hydrogen charging current densities: (**a**) uncharged with hydrogen, (**b**) 10 mA/cm^2^, (**c**) 15 mA/cm^2^, (**d**) 20 mA/cm^2^.

Figure 8 shows the relationship between internal friction (Q^−1^) and temperature spectra of the experimental steel under different hydrogen charging current densities. By combining the peak temperature and peak frequency of each internal friction peak, the activation energy of each peak can be calculated according to Equation (7).

(7) $$H=\frac{R\cdot T_{m}\cdot ln\left(\frac{k_{B}\cdot T_{m}}{h\cdot F_{m}}\right)}{N_{A}}\cdot 6.25\times 10^{18}+T_{m}\cdot \Delta S$$

where *H* is the activation energy of the internal friction peak, in eV; *R* is the gas constant; *T_m_* is the peak temperature of the corresponding peak, in K; *k_B_* is Boltzmann’s constant; *h* is Planck’s constant; *F_m_* is the peak frequency, in Hz; Δ*S* is the entropy change, which is 1.1 × 10^−4^ eV/K [33,34].

The internal friction-temperature spectra obtained from internal friction experiments on the specimens under different hydrogen charging current densities, with background internal friction subtracted, are shown in Figure 8. To more clearly and intuitively characterize the changes and underlying mechanisms of each internal friction peak in the internal friction-temperature spectra, four peaks are observed both before and after hydrogen charging. These peaks are distinguished based on their peak temperatures from low to high and are referred to as P1, P2, P3, and P4, respectively. The activation energies and related parameters of the internal friction peaks calculated according to Equation (7) are summarized in Table 5.

As clearly shown in Figure 8, the P1 peak is not observed in the experimental steel under non-hydrogen-charged conditions. After hydrogen charging, the P1 peak appears, and its peak height gradually increases with rising hydrogen charging current density. The activation energy decreases from 0.68 eV to 0.598 eV, with the peak temperature ranging between −29.6 °C and −39.3 °C. The P2, P3, and P4 peaks are observed both before and after hydrogen charging. The activation energy of the P2 peak decreases from 0.871 eV to 0.862 eV, while that of the P3 peak decreases from 1.021 eV to 1.002 eV. The activation energy of the P4 peak decreases from 1.519 eV to 1.477 eV. Overall, the peak heights of the P1, P2, P3, and P4 peaks increase with higher current density.

The P1 peak is a hydrogen-induced internal friction peak caused by the diffusion of interstitial hydrogen atoms. Its physical essence lies in the lattice migration behavior of interstitial hydrogen. According to its peak temperature and activation energy, the P1 peak is identified as resulting from the diffusion process of hydrogen atoms at interstitial sites and vacancies, driven by thermal stress. It is also considered a mixed peak consisting of three hydrogen-induced internal friction peaks: the Snoek peak, the SKK peak, and the Hcwp peak [33]. During the 8-h hydrogen charging process, the current density increased from 0 to 20 mA/cm^2^. This increase in current density significantly alters the hydrogen permeation characteristics of the material. As the hydrogen concentration rises, hydrogen enrichment increases the proportion of mobile hydrogen. Consequently, the activation energy of the material gradually decreases, and the peak value increases. The P1 peak is attributed to a Snoek peak resulting from the diffusion of interstitial hydrogen atoms, with its physical origin rooted in the lattice migration behavior of interstitial hydrogen. During the 8-h hydrogen charging process, as the current density increases from 0 to 20 mA/cm^2^, the rise in current density significantly alters the hydrogen permeation characteristics of the material. With increasing hydrogen concentration, the enrichment of hydrogen elevates the proportion of mobile hydrogen, thereby gradually reducing the activation energy of the material and raising the peak height.

The P2 peak is a Snoek peak resulting from the diffusion of interstitial carbon atoms. Before and after hydrogen charging, the influence of current density on the activation energy and peak temperature is minimal, with the peak temperature ranging from 62.4 °C to 59.8 °C and the activation energy between 0.868 eV and 0.862 eV. Due to its smaller atomic radius and lower relative atomic mass, hydrogen atoms exhibit a significantly higher migration rate than carbon in iron-based alloys. When the hydrogen concentration increases, this hydrogen-induced effect markedly inhibits the diffusion behavior of carbon atoms within the lattice structure. However, as hydrogen atoms gradually occupy interstitial sites in the lattice, the overall atomic diffusion capacity is enhanced, leading to a slight reduction in the activation energy required for carbon atom diffusion [33].

The P3 peak is a Snoek-Kê-Köster (SKK) internal friction peak arising from the interaction between interstitial carbon atoms and dislocations. Before and after hydrogen charging, the activation energy of the P3 peak remains in the range of 1.021 eV to 1.002 eV, with peak temperatures between 120.5 °C and 112.2 °C. This behavior may be attributed to the formation of different interstitial atom pairs (such as C–H, C–C, or H–H pairs), which can be bound together via covalent bonding. Under the influence of a stress field, these co-occupying atom pairs interact with dislocations to form Cottrell atmospheres, making dislocation motion more difficult and increasing friction and misfit energy between dislocations and surrounding atoms. As the covalent interactions strengthen, more atom pairs can be activated or move under lower energy conditions, which may cause the SKK peak to shift toward lower temperatures.

The P4 peak corresponds to a grain boundary relaxation peak, with an activation energy ranging from 1.519 eV to 1.477 eV and a peak temperature between 297.2 °C and 280.5 °C. Under varying hydrogen charging current densities, the activation energy of the P4 peak gradually decreases, and the extent of reduction is greater than that observed for the previous two peaks. As the hydrogen charging current density increases, the number of phase-interface dislocations rises significantly. These dislocations act as trapping centers for hydrogen atoms, enhancing the hydrogen absorption capacity of the alloy. Hydrogen atoms become more likely to segregate along grain boundaries and phase interfaces, thereby improving the deformability of the material. Simultaneously, the introduction of hydrogen atoms alters the microstructure and stress state of the material, creating an unstable high-energy state that provides a driving force for grain boundary movement and promotes grain boundary relaxation.

## 4. Conclusions

This study systematically investigated the hydrogen trapping and embrittlement in an ultra-high-copper steel (6.01 wt.% Cu) under varying hydrogen charging current densities. The investigation employed microstructure characterization, slow strain rate tensile testing, electrochemical hydrogen permeation, and internal friction measurements. The main conclusions are as follows:

(1) The hydrogen charging current density significantly influences mechanical properties and hydrogen embrittlement susceptibility. In the ultra-high-copper steel, the increased copper content results in a higher density of Cu-rich precipitates at grain boundaries and within grains, which act as effective irreversible hydrogen traps, thereby suppressing hydrogen diffusion and enhancing hydrogen embrittlement resistance.
(2) As the hydrogen charging current density increases, the hydrogen permeation flux and effective diffusion coefficient increase significantly, while the breakthrough lag time shortens. The marked increase in irreversible hydrogen trap density under high-current conditions indicates that hydrogen atoms are more readily trapped by grain boundaries, dislocations, and precipitate interfaces, which slows hydrogen diffusion and mitigates hydrogen-induced embrittlement.
(3) Internal friction analysis reveals that hydrogen introduction promotes atomic migration and grain boundary motion, as evidenced by the appearance of a hydrogen Snoek peak and the decreased activation energies of carbon diffusion, carbon-dislocation interaction, and grain boundary relaxation peaks. These findings confirm that microscopic defects play a critical role in hydrogen trapping and release.

This study demonstrates that increasing copper content to 6.01 wt.% significantly enhances the hydrogen embrittlement resistance of low-carbon steel by promoting the formation of Cu-rich precipitates that act as effective irreversible hydrogen traps. These findings provide valuable guidance for the design of high-performance, hydrogen-resistant steel materials for marine engineering and other hydrogen-rich service environments.

## Figures and Tables

**Table 1:** Chemical composition of the tested steel (wt.%).

C	Cu	Ni	Mn	Cr	Si	Ti
0.036	6.01	2.70	0.97	0.48	0.20	0.0089

**Table 2:** Mechanical properties of the tested steel before and after hydrogen charging.

Hydrogen Charging Conditions	Yield Strength/MPa	Tensile Strength/MPa	Elongation/%	Hydrogen Embrittlement Sensitivity/%
Uncharged	916 ± 8	960 ± 6	22.8 ± 1.2	-
10 mA/cm^2^	909 ± 7	956 ± 5	17.3 ± 0.9	24.1
15 mA/cm^2^	888 ± 10	926 ± 8	16.8 ± 0.7	26.3
20 mA/cm^2^	840 ± 12	861 ± 9	13.1 ± 0.8	42.5

**Table 3:** Quantitative analysis of brittle fracture area fraction under different hydrogen charging conditions.

Hydrogen Charging Conditions	Brittle Fracture Area Ratio/%	Estimated Error/±%
Uncharged	0	0
10 mA/cm^2^	5	3
15 mA/cm^2^	12	3
20 mA/cm^2^	30	3

**Table 4:** Hydrogen permeation kinetic parameters of test steel under different hydrogen charging currents.

Parameter	10 mA/cm^2^		15 mA/cm^2^		20 mA/cm^2^	
	1st Permeation	2nd Permeation	1st Permeation	2nd Permeation	1st Permeation	2nd Permeation
*t_lag_* (s)	862.2	836.35	745.8	443.4	1038.6	739.2
*J_ss_* (mol/cm^2^/s)	6.7 × 10^−8^	6.57 × 10^−8^	9.34 × 10^−8^	8.76 × 10^−8^	1.23 × 10^−7^	1.15 × 10^−7^
*D_eff_* (cm^2^/s)	1.93 × 10^−6^	3.33 × 10^−6^	2.23 × 10^−6^	3.76 × 10^−6^	2.76 × 10^−6^	4.54 × 10^−6^
*C*_0_ (mol/cm^3^)	3.47 × 10^−3^	1.97 × 10^−3^	4.19 × 10^−3^	2.33 × 10^−3^	4.46 × 10^−3^	2.5 × 10^−3^
*N_T_* (cm^−3^)	3.36 × 10^22^	3.09 × 10^22^	3.5 × 10^22^	1.13 × 10^22^	8.97 × 10^22^	4.21 × 10^22^
*N_ir_* (cm^−3^)	2.7 × 10^21^		2.37 × 10^22^		4.76 × 10^22^	

**Table 5:** Internal friction peak parameters of copper-containing mild steel with different current densities.

Hydrogen Charging Current Density *I*/(mA/cm^2^)	Internal Friction Peak	Peak Temperature *T_m_*/°C	Frequency *F_m_*/Hz	Activation Energy *H*/eV
Uncharged	P2	64.1	1.88	0.871
	P3	120.5	1.868	1.021
	P4	297.2	1.824	1.519
10	P1	−29.6	1.91	0.68
	P2	62.4	1.849	0.868
	P3	117.2	1.841	1.014
	P4	291.9	1.796	1.506
15	P1	−33.2	1.669	0.621
	P2	61.7	1.648	0.868
	P3	114.5	1.633	1.013
	P4	286.6	1.599	1.498
20	P1	−39.3	1.779	0.598
	P2	59.8	1.771	0.862
	P3	112.2	1.761	1.002
	P4	280.5	1.715	1.477

## Data Availability

The original contributions presented in the study are included in the article, further inquiries can be directed to the corresponding author.
